# A protocol for displaying viral envelope glycoproteins on the surface of vesicular stomatitis viruses

**DOI:** 10.1016/j.xpro.2020.100209

**Published:** 2020-12-09

**Authors:** Touraj Aligholipour Farzani, Angela Chov, Alon Herschhorn

**Affiliations:** 1 Division of Infectious Diseases and International Medicine, Department of Medicine, University of Minnesota, Minneapolis, MN 55455, USA; 2 Microbiology, Immunology, and Cancer Biology Graduate Program, University of Minnesota, Minneapolis, MN 55455, USA; 3 The College of Veterinary Medicine Graduate Program, University of Minnesota, Minneapolis, MN 55455, USA; 4 Institute for Molecular Virology, University of Minnesota, Minneapolis, MN 55455, USA

**Keywords:** Cell culture, Cell-based assays, Immunology, Molecular biology

## Abstract

We describe the production of single-cycle (sc) and replication-competent recombinant vesicular stomatitis viruses (rcVSVs) displaying heterologous envelope glycoproteins (Envs) on their surface. We prepare scVSVs by transiently expressing HIV-1 Envs or SARS-CoV-2 spike followed by infection of the cells with scVSV particles, which do not carry the *vsv-g* gene. To prepare rcVSVs, we replace the vsv-g with a specific *env*-encoding gene, transfect cells with multiple plasmids for production of the genomic RNA and viral proteins, and rescue replication-competent viruses.

## Highlights

• Heterologous, viral envelope glycoproteins can be displayed on recombinant VSV
• Recombinant VSVs allow study of the biology of viral entry of different viruses
• Recombinant VSVs can be used to measure virus neutralization and as vaccines

## Graphical abstract

**Figure:**
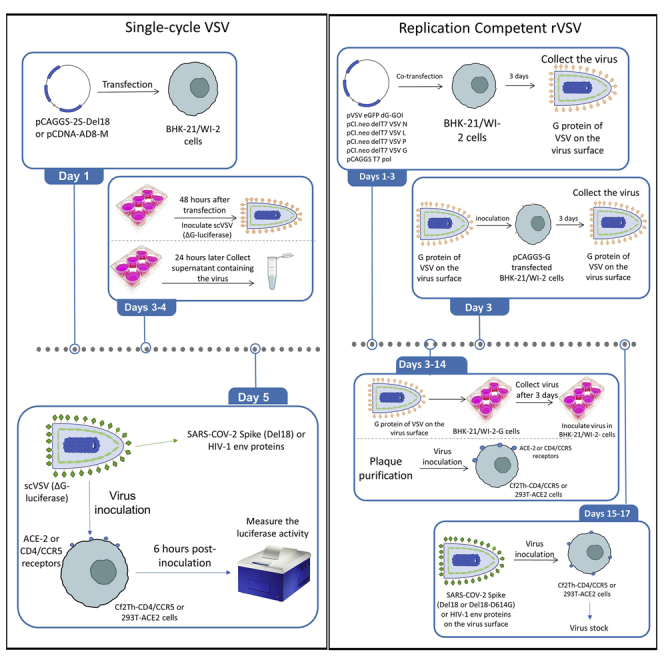

## Before you begin

### Introduction

VSV belongs to the Rhabdoviridae family (genus vesiculovirus) and contains a non-segmented, negative-sense, single-stranded RNA genome. VSV expresses five major proteins during viral replication: glycoprotein (G), matrix (M), nucleoprotein (N), large protein (L), and phosphoprotein (P). VSV-G mediates viral entry by binding to target cell and facilitating the fusion of the viral membrane with the host endosomal membrane following endocytosis.

**Figure 1:**
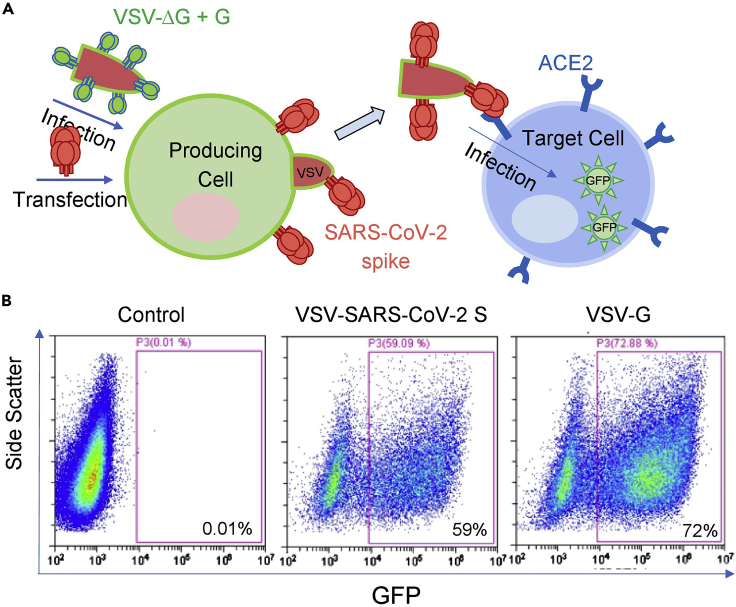
scVSV-2S del18 preparation and infection (A) A scheme for scVSV preparation. SARS-Cov-2 spike with an 18-amino acid deletion at the C-terminal is pseudotyped on the surface of VSV particles that carry the *gfp* reporter gene. scVSV-SARS-2-S del18 pseudoviruses can infect 293T-ACE2 target cells. (B) scVSV-2S del18 or the VSV control displaying the VSV-G protein were used to infect 293T-ACE2 cells and GFP expression was analyzed by flow cytometry.

In this protocol, we describe a method for preparation of single-cycle VSVs in which the *vsv-g* gene is deleted from the viral genome and heterologous envelope glycoproteins (Envs or Spike) are provided in trans during the virus production in BHK-21/WI-2 cells (Whitt, 2010). These Envs may have to be truncated and, in some cases, fused to the VSV-G cytoplasmic tail to allow efficient packaging of the Envs on the VSV surface. scVSVs can enter permissive cells, which express the target receptor and interacts with the Envs (for example, SARS-CoV-2 spike; Hoffmann et al., 2020), but they cannot replicate in the target cells as they lack an *env* gene to produce VSVs that could mediate viral entry (Kapadia et al., 2008). scVSVs typically carry reporter genes such as *green fluorescence protein* (*gfp*) or *firefly luciferase (fluc)* that allow to evaluate the efficiency of infection (Figure 1). In addition, we describe a method for preparation of replication-competent VSV in which heterologous envelope glycoproteins genes are introduced into the VSV genome, replacing the native vsv-g gene. Preparation of rcVSVs is relatively long but once these viruses are rescued, they can be routinely amplified in target cells.

Recombinant VSVs can be used as alternatives for the study of Envs of viruses that typically require BSL3 and BSL4 environments for processing and growth. Notably, VSVs can be used in many different in vitro experimental systems (Tani et al. 2012) including neutralization assays (Schmidt et al. 2020; Furuyama et al., 2020), virus adaptation to different environments or ligands (Baum et al. 2020), as probes to identify broadly neutralizing antibodies (Jia et al., 2020), and for in vivo immunogen delivery for vaccine development (Jiang et al., 2006; Rose et al. 2001).

### Biosafety

The National Institutes of Health (NIH) guidelines classify the following vesicular stomatitis virus non-exotic strains as Risk Group 2 agents: VSV-Indiana 1 serotype strains (e.g., Glasgow, Mudd- Summers, Orsay, San Juan) and VSV-New Jersey serotype strains (e.g., Ogden, Hazelhurst). Research involving VSV is typically done in biosafety level 2 (BSL-2) environment.

We routinely use scVSVs carrying a reporter gene under BSL2 practices but rVSVs are prepared and used in a BSL2+ facility due to potential tropism change of a replication-competent virus. Nevertheless, an attenuated rcVSV displaying the Ebola glycoprotein is approved by the FDA as a preventive vaccine for the disease caused by Zaire ebolavirus (ERVEBO [Ebola Zaire Vaccine, Live]; https://www.fda.gov/media/133748/download). Thus, research work involving rcVSV should be done according to federal and institutional guidelines and regulations.

### Cell maintenance

**Timing: 2–3 days**

1. Remove a frozen cell vial from the liquid nitrogen tank and place the tube immediately in a 37°C water bath until cells are thawed. Transfer the cells to a 15-mL tube with 10 mL DMEM medium, centrifuge at 100–200 × *g* for 6 min, discard the supernatant, and suspend the cells in fresh DMEM medium.
2. Maintain each cell line in the recommended medium:
  a. BHK-21/WI-2 cells. Dulbecco’s Modified Eagle Medium (DMEM) containing 10% fetal bovine serum (FBS), 100 μg/mL streptomycin, and 100 units/mL penicillin.
  b. Cf2Th-CD4/CCR5 cells. DMEM containing 10% FBS, 100 μg/mL streptomycin, 100 units/mL penicillin, and supplemented with 400 μg/mL G418, and 200 μg/mL hygromycin B.
  c. 293T-ACE2 cells: DMEM containing 10% FBS, 100 μg/mL streptomycin, 100 units/mL penicillin, and supplemented with 1 μg/mL puromycin.
3. Split the cells every 2–3 days at a ratio of 1/3 to 1/5. We typically split the cells at < 90% confluency (every 2–3 days) using the following protocol (Ratnapriya et al. 2020):
  a. Remove the media from the flask, followed by washing once with PBS.
  b. Add 1–2 mL of dissociation reagent (TrypLE or StemPro Accutase) to the adhered cells and incubate until cells are detached (typically < 5 min).
  c. Add 10 mL of DMEM to the flask, collect the detached cells, and mix slowly by gentle pipetting up and down.
  d. Dilute the cell suspension as necessary, transfer the required volume of cells to a new flask and add selection antibiotics if needed. BHK-21/WI-2 cells grow fast compared to other cells used in this study.

***Note:*** All cells should be passaged at least twice and should show healthy morphology before using them. (ATCC recommends passaging a culture no more than 8 to 10 passages or 2 months (https://lgcstandards-atcc.org/support/faqs/6fbf9/Maximum%20Passage%20Number-4.aspx)).

***Note:*** All cells should be tested for mycoplasma and they are typically maintained in vented culture flasks at 37°C incubated with 5% CO_2_.

***Alternatives:*** GHOST CCR5+ Cells (Hi-5) can be used instead of the Cf2Th-CD4/CCR5. These cells are expressing CD4 and relatively high levels of CCR5.

***Alternatives:*** Vero-E6 (VERO C1008; ATCC CRL-1586) can be used instead of 293T-ACE2 cells.

### Plasmids

4. For generation of scVSVs, we use the following Envs (or Spike)-expression plasmids:
  a. *SARS-CoV-2 Spike:* **pCAGGS-2S-del18** - A plasmid for expression of SARS-CoV-2 spike with an 18-amino acid deletion of the cytoplasmic tail
  b. *HIV-1 Envs*: **pCDNA-AD8-M-G**_**CT**_ - A plasmid for expression of a codon-optimized ectodomain and transmembrane regions of HIV-1_AD8_ Env fused to the cytoplasmic tail of VSV-G (similar to the chimeric Env described in Liberatore et al. 2019)
5. For generation of rcVSVs we use the following plasmids:
  a. **pVSV eGFP dG** - A plasmid for transcribing the antisense genomic RNA of VSV in producing cells by the T7 RNA polymerase. For DNA preps, the plasmid should be propagated in bacteria at 30°C.
  b. VSV-protein expression plasmids:
    i. **pCI.neo delT7 VSV N**
    ii. **pCI.neo delT7 VSV L**
    iii. **pCI.neo delT7 VSV P**
    iv. **pCI.neo delT7 VSV G**
  c. **pCAGGS T7 pol**, T7-expression plasmid
  d. HIV-1 Env or SARS-CoV-2 Spike expressing plasmids (see above)

## Key resources table

**Table:** 

REAGENT or RESOURCE	SOURCE	IDENTIFIER
**Chemicals, peptides, and recombinant proteins**		

AddaVax adjuvant	Invivogen	Cat# vac-adx-10
Adenosine 5′-triphosphate disodium salt hydrate (ATP)	Sigma	Cat# A26209-10G
PfuUltra II Fusion High-fidelity DNA Polymerase (Agilent)	Agilent	Cat# 600672
Agar	BD	Cat# 281230
Ampicillin sodium salt	Sigma	Cat# A9518-5G
Bovine serum albumin (BSA)	Sigma	Cat# A2153-100G
Carbenicillin disodium salt	VWR Life Science	Cat# J358-1G
D-Luciferin phosphate (chemical name: D- (-)-2-(6′-hydroxy-2′-benzothiazolyl)- thiazoline-4-carboxylic acid)	BD Biosciences	Cat# 556879
Deoxynucleotide (dNTP) solution mix	NEB	Cat# N0447S
Dimethyl sulfoxide (DMSO)	Sigma	Cat# D2438-10ML
Dithiothreitol (DTT)	Sigma	Cat# 43816-10ML
Dulbecco’s modified Eagle’s medium (DMEM)	Gibco	Cat# 11965-084
Dulbecco’s phosphate buffered saline (PBS)	Sigma	Cat# D8537-500ML
Effectene transfection reagent	Qiagen	Cat# 301425
Ethylene diamine tetraacetic acid (EDTA)	Promega	Cat# V4231
Fetal bovine serum (FBS)	Gibco	Cat# 10437-010
Geneticin G418 sulfate	Invitrogen	Cat# 10131027
Glucose	Alfa Aesar	Cat# A16828
Glycerol	Fisher Chemical	Cat# G33-500
HEPES	Sigma	Cat# H4034-25G
Hydrogen peroxide solution (30% w/w)	Sigma	H1009-100ML
Hygromycin B	Invitrogen	Cat# 10687010
Puromycin dihydrochloride	Thermo Fisher	Cat# A1113802
Magnesium sulfate (MgSO_4_)	Sigma	M1880-500G
NEBuilder HiFi DNA Assembly Master Mix	NEB	Cat# E2621L
Penicillin-streptomycin (PenStrep)	Gibco	Cat# 15140-122
Phosphoric acid (H_3_PO_4_)	Sigma	Cat# 466123-25G
Potassium chloride (KCl)	Sigma	Cat# P5405-250G
Potassium phosphate dibasic (K_2_HPO_4_)	Sigma	795496-500G
Potassium phosphate monobasic (KH_2_PO_4_)	Sigma	795488-500G
Sodium chloride (NaCl)	Sigma	Cat# S5886-5KG
Sodium hydroxide (NaOH)	Sigma	Cat# 58045-500G
Sodium phosphate dibasic (Na_2_HPO_4_)	Sigma	Cat# S5136-100G
Soluble SARS-CoV-2 spike protein	This experiment	N/A
SuperScript II reverse transcriptase	Thermo Fisher	Cat# 18064022
StemPro accutase	Gibco	Cat# A11105-01
*trans*-1,2-Diaminocyclohexane-N,N,N′,N′-tetraacetic acid monohydrate (DCTA)	Sigma	Cat# 319945-25G
Tris base	Fisher BioReagents	Cat# BP152-1
Triton X-100	Sigma	Cat# X100-100ML
TrypLE Express (-) phenol red	Gibco	Cat# 12604-021
Tryptone	BD	Cat# 211705
Tween-20	BIO-RAD	170-6531
Yeast extract	BD	Cat# 212750

**Experimental models: cell lines**		

Cf2Th CD4/CCR5 cells	Laboratory of Joseph Sodroski	Parental Cf2Th cells are from ATCC (CRL-1430)
HEK 293T-ACE2 cells	Laboratory of Fang Li	N/A
BHK-21/WI-2	Kerafast	Cat# EH1011

**Recombinant DNA**		

pVSV eGFP dG (full sequence of VSV with the vsv-g gene deleted (ΔG); transcribed from a T7 promoter)	Addgene	Plasmid #31842
pCI.neo delT7 VSV N (VSV N-expression plasmid)	Laboratories of Ryan Langlois and Benjamin tenOever	N/A
pCI.neo delT7 VSV L (VSV L-expression plasmid)	Laboratories of Ryan Langlois and Benjamin tenOever	N/A
pCI.neo delT7 VSV P (VSV P-expression plasmid)	Laboratories of Ryan Langlois and Benjamin tenOever	N/A
pCI.neo delT7 VSV G (VSV G-expression plasmid)	Laboratories of Ryan Langlois and Benjamin tenOever	N/A
pCAGGS T7 pol (T7 enzyme expression plasmid)	Laboratories of Ryan Langlois and Benjamin tenOever	N/A
pCAGGS-2S-del18 (expression of SARS-CoV-2 Spike with an 18-amino acid deletion at the C terminus)	Herschhorn lab. Original pCG1-SARS-2-S plasmid is from Laboratory of Stefan Pohlmann (Hoffmann et al. 2020)	N/A
pCDNA-AD8-M-G_CT_ (expression of a chimeric Env: HIV-1_AD8_ ecto- and transmembrane domains fused to VSV-G cytoplasmic tail)	Herschhorn lab	N/A
pCAGGS-G (VSV-G expressing plasmid)	Kerafast	Cat# EH1017

**Bacterial and virus strains**		

scVSV-G.luc (pseudotyped ΔG-luciferase (G∗ΔG-luciferase) rVSV)	Kerafast	Cat# EH1020-PM
scVSV-G.gfp (pseudotyped ΔG-GFP (G∗ΔG-GFP) rVSV)	Whitt lab. Available also from Kerafast	Cat# EH1019-PM

**Software and algorithms**		

Gen5	BioTek Instruments	Version 2.09
MikroWin 2000 Lite	Berthold Technologies GmbH	Id. Nr. 37854-304
Prism	GraphPad	https://www.graphpad.com/
SnapGene	GSL Biotech LLC	https://www.snapgene.com/

**Experimental models: organisms/strains**		

Mouse: BALB/c	Charles River Laboratories (USA)	N/A

**Oligonucleotides**		

AD8-M-G_CT_-F: TCGATCTGTTTCC TTGACACGCGTTACGATA TGAAGGTGAAGGGCATCCG	Thermo Fisher Scientific	N/A
AD8-M-G_CT_-R: GGTTCAAACATG AAGAATCTGTTGTGCAGGTTACT TTCCAAGTCGGTTCATCTCTATGT	Thermo Fisher Scientific	N/A
2S (del18)-VSV-F: TCGATCTGTTTCC TTGACACGCGTTACGATATGTTTC TGCTGACCACCAAGC	Thermo Fisher Scientific	N/A
2S (del18)-VSV-R: GGTTCAAACA TGAAGAATCTGTTGTGCAGGTT ACTTGCAGCAGCTGCCACA	Thermo Fisher Scientific	N/A
Random Hexamers (100 μL)	Qiagen	Cat No./ID: 79236

**Other**		

Tissue Culture 96-well Microplate (Luminometer plates)	Greiner bio-one	Cat# 655083
Microplate luminometer	Berthold Technologies GmbH	Centro LB 960 XS3
Spectrophotometer	BioTek	SYNERGY/H1 microplate reader

**Critical commercial assays**		

Wizard SV Gel and PCR Clean-Up System	Promega	Cat# A9281
NucleoSpin RNA Virus Kit	Macherey-Nagel	REF 740956.50

## Materials and equipment

### Transfection, luciferase lysis buffer, firefly luciferase assay buffer, luciferin substrate solution, and vacuum system

Solutions and equipment are described in detail in Ratnapriya et al. 2020.

## Step-by-step method details

### Preparation of single-cycle vesicular stomatitis viruses (scVSVs)

**Timing: 6 days**

In many cases, scVSVs allow to study Envs of pathogenic viruses, which require BSL-3 and BSL-4 environments, under BSL-2 practices. As an example, we describe the preparation of scVSVs that display the HIV-1_AD8_ Env and SARS-CoV-2 spike. The HIV Envs and SARS-CoV-2 spike have been the subject of intensive research investigations (Bar et al., 2016; Flemming et al., 2018; Harris et al. 2020; Herschhorn et al., 2011, 2014, 2016; Hoffmann et al., 2020; McLellan et al., 2011; Pancera et al., 2014; Parrish et al., 2013; Riva et al., 2020; Wrapp et al., 2020).

1. **Day 1:** Detach BHK-21/WI-2 cells using StemPro Accutase Cell Dissociation Reagent and count viable cells using trypan blue exclusion dye.
2. Add 5 × 10^5^ cells in 2 mL of DMEM medium to each well of 6-well plate and incubate the cells for 24 h at 37°C and 5% CO_2_ concentration in a tissue culture incubator.
3. **Day 2:** One hour before transfection, change the medium of each well and add 2 mL of medium without FBS and antibiotic

**CRITICAL:** This time schedule ensures pH equilibration.

4. Transfect the cells with 0.4 μg of Env-expressing plasmid (e.g., pCAGGS-2S-del18, or pCDNA-AD8-M-G_CT_) using Effectene according to the manufacturer’s instructions (https://www.qiagen.com/us/products/discovery-and-translational-research/functional-and-cell-analysis/transfection/effectene-transfection-reagent/#orderinginformation).

***Alternatives:*** Calcium phosphate transfection can be used instead of Effectene [The calcium phosphate transfection mix is prepared by mixing 2× HEPES Buffer (50 mM HEPES, 10 mM KCl, 12 mM Glucose, 280 mM NaCl, 1.5 mM Na_2_HPO_4_; pH 7.2), 2 M CaCl_2_, and DNA as described in Ratnapriya et al. 2020]. In our experience, Effectene was more efficient than calcium phosphate for transfection of BHK-21/WI-2 cells.

**CRITICAL:** We recommend incubating the transfected cells for two days before following the next step. There is no need to change the media as Effectene does not show any significant cytotoxic effects to BHK-21/WI-2 cells during incubation.

5. **Day 4: Infection the transfected cells with scVSV-G (ΔG-luciferase).** Remove the media from attached transfected cells and wash 3 times with PBS to remove dead cells. The transfected cells are typically ∼90% confluent at the time of virus inoculation.
6. Add 500 μL medium without FBS to each well.
7. Add scVSV-G (ΔG-luciferase (G∗ΔG-luciferase) rVSV) at multiplicity of infection (MOI) of 3 to each well. The scVSV-G viruses display the VSV-G protein on their surface but carry VSV genome in which the *vsv-g* gene was deleted. Thus, new progeny scVSVs that are produced in the BHK-21/WI-2 cells will contain the deleted genome and display the heterogenous Env that is expressed from a separate Env-expressing plasmid.
8. Incubate at 37°C for 1 h with a frequent gentle shake every 15 min.
9. Remove the free scVSV-G (ΔG-luciferase (G∗ΔG-luciferase) rVSV) virus from the cells and wash once with PBS.
10. Add 2 mL of medium without FBS containing 1/1000 dilution of anti-VSV-G antibody (we usually use clone 8G5F11 from Millipore; catalog number MABF2337-100UG). The addition of antibody ensures that potential traces of VSV-G on the progeny scVSVs are not able to mediate viral entry.
11. Incubate the infected cells for 24 h in the tissue culture incubator at 37°C and 5% CO_2_ concentration. At this step infected cells will produce scVSVs displaying the Env that was transfected on day 2 (step 4). Transfer the scVSV-containing supernatant to a tube and centrifuge at 500 × *g*; collect the virus and store in aliquots at −80°C.

***Note:* For days 5–6,** titer the scVSVs on target cells based on their reporter gene. Target cells should express the receptor for the specific Envs that are displayed on the scVSVs. We typically use 293T-ACE2 and Cf2Th-CD4/CCR5 cells for SARS-CoV-2 spike and HIV-1 Env scVSVs, respectively. VSV has a short life cycle and reporter protein expression can be measured between 6- and 24-h post infection.

***Note:*** It is recommended to test different time points to detect the kinetics and saturation of the assay.

12. **Day 5:** Seed 2 × 10^4^ cells/well target cells in 96-well white luminometer plate.
13. **Day 6:** Serially dilute the scVSVs 10-fold in DMEM ranging from 10^−1^ to 10^−8^.
14. Aspirate the media of each well in the 96-well plate and add 100 μL of the diluted scVSVs to the wells in triplicate. Include a cell control group with no virus to calculate the background level in the luciferase assay. Incubate the infected cells in the tissue culture incubator at 37°C and 5% CO_2_ concentration for 6 h.
15. Aspirate the media completely and add 31 μL of lysis buffer to each well.
16. Incubate the plate for 20 min at 20°C–25°C and measure the firefly luciferase activity using a luminometer as described in Ratnapriya et al. 2020.

***Note:*** We also prepare scVSVs that express GFP upon infection by using the same protocol but with scVSV.gfp (Figure 1).

***Note:*** Luciferase activity can be also detected in the supernatant of infected cells but the readout from lysed cells is usually higher than the readout from the supernatant.

### Preparation of plasmids for the rescue of replication-competent vesicular stomatitis viruses (rcVSVs)

**Timing: 4 days**

The production of rcVSVs is based on the pVSV eGFP dG plasmid (available from Addgene), which contains the complete VSV genome with a deletion of the *vsv-g* gene and a convenient multiple cloning site between the *matrix* (M) and *polymerase* (L) genes that includes the MluI and NotI restriction sites. A heterologous *env* gene can be introduced by standard cloning procedures or by Gibson assembly. Primers for Gibson assembly typically contain 20–25 bp overlapping end sequences that match the plasmid sequences at the point of assembly. To generate rcVSVs, the resulting plasmid, which contains the VSV genome and heterologous env, is co-transfected with an additional 5 plasmids encoding for viral proteins and the T7 RNA polymerase (Lawson et al., 1995; Langlois et al. 2012). rcVSVs are rescued from the transfected cells and can be used for a variety of applications (Bresk et al., 2019; Case et al., 2020; Liberatore et al., 2019).

17. **Day 1:** Design the primers for Gibson assembly (Table 1). These can be done by using online tools such as NEBuilder Assembly Tool or manually using any DNA editor program.

**Table 1:** Primers used for amplification of HIV-1 *env* and SARS-CoV-2 *spike*

Primers^a^	Sequence	Target	Length
AD8-M-G_CT_	F: tcgatctgtttccttgacacgcgttacgat ATGAAGGTGAAGGGCATCCG R: ggttcaaacatgaagaatctgttgtgcagg TTACTTTCCAAGTCGGTTCATCTCTATGT	HIV-1_AD8_ env	2,127 bp
2S (del18)-VSV	F: tcgatctgtttccttgacacgcgttacgat ATGTTTCTGCTGACCACCAAGC R: ggttcaaacatgaagaatctgttgtgcagg TTACTTGCAGCAGCTGCCACA	SARS-CoV-2 spike gene	3,795 bp

a Underlying sequences are homologous to sequences in the target plasmid used for Gibson assembly.
18. **Day 2 (day in which primers are available):** Amplify the gene of interest by PCR using a high-fidelity DNA polymerase to reduce potential errors. We typically design primers with a melting temperature (Tm) of 60°C and use PfuUltra II Fusion High-fidelity DNA Polymerase (Agilent) according to the manufacturer`s instructions (https://www.agilent.com/en/product/polymerase-chain-reaction-(pcr)/pcr-enzymes-reagents/high-fidelity-gc-rich-target-dna-polymerases-for-pcr/pfuultra-ii-fusion-high-fidelity-dna-polymerase-785916).
19. Separate the PCR products on 0.8%–1.2% agarose gel and extract the DNA with the correct size using a commercial gel-extraction kit (e.g., Wizard SV Gel and PCR Clean-Up System from Promega). This step is not necessary for Gibson assembly, but it allows to verify the correct size of the amplified DNA and, according to our experience, can increase the efficiency of the reaction.
20. Set up the Gibson assembly reaction according to the manufacturer’s instructions (https://www.neb.com/products/e2621-nebuilder-hifi-dna-assembly-master-mix#Product%20Information). Incubate the reaction mixture at 50°C for 15 min, transform the reaction products into bacteria, and incubate 16–20 h at 30°C on LB agar plates containing 50 μg/mL of carbenicillin.
21. **Days 3–4:** Select 2–3 well-isolated and round colonies, inoculate in 2 mL of LB broth containing 50 μg/mL of ampicillin and grow the bacteria 16–20 h at 30°C in a shaker incubator.
22. The next day extract the plasmids using commercial minipreps (e.g., Macherey-Nagel). Analyze the plasmids by restriction enzymes to verify the present of the env gene and sequence the gene. Figure 2 shows the resulting plasmid map after cloning.

**Figure 2:**
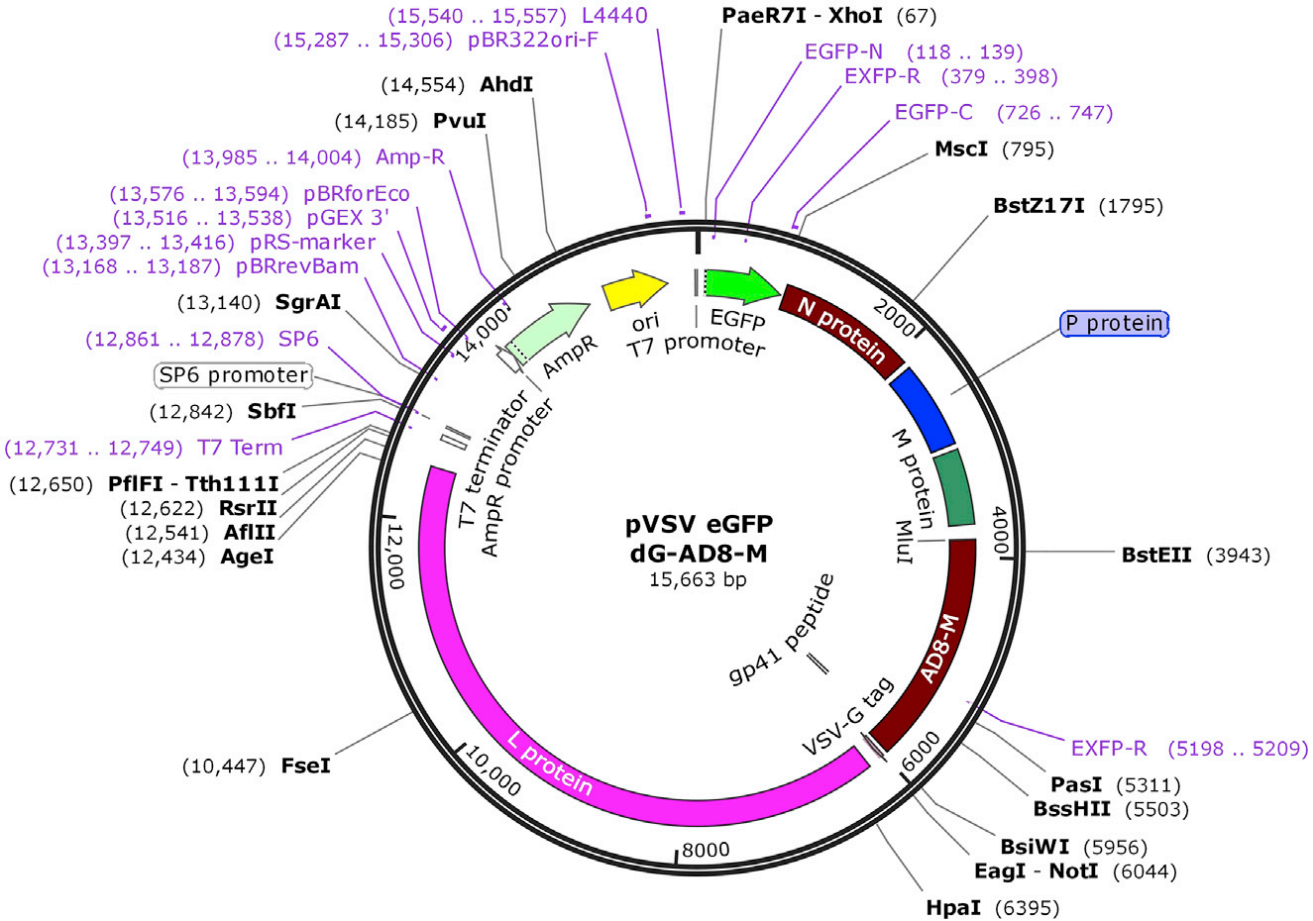
pVSV eGFP dG AD8-M plasmid map The HIV-1_AD8_*env* gene was introduced between VSV *m* and *l* genes in the original pVSV eGFP dG plasmid (available from Addgene). A similar plasmid was constructed by replacing the *AD8-M* gene with the SARS-CoV-2 *spike* gene to generate pVSV eGFP dG 2S-Del18.

### Production of replication-competent vesicular stomatitis viruses (rcVSVs)

**Timing: 15 days**

23. **Day 1**: Seed 4 × 10^5^ BHK-21/WI-2 cells in a 6-well plate.
24. **Day 2:** Change the media (2 mL) 1 h before the transfection. Do not include fetal bovine serum (FBS) and antibiotics in the media.
25. Prepare the VSV and supporting plasmids at the ratio specified below and transfect the cells using Effectene. To increase DNA transfection without cytotoxicity of reagents, we typically use a total of 0.85 μg DNA, 3.2 μL enhancer, and 10 μL Effectene.

**Table:** 

pVSV eGFP dG-env^a^	200 ng
pCI.neo delT7 VSV N	50 ng
pCI.neo delT7 VSV L	25 ng
pCI.neo delT7 VSV P	125 ng
pCI.neo delT7 VSV G	200 ng
pCAGGS T7 pol	250 ng

a pVSV eGFP dG-env, pVSV eGFP dG-AD8-M-G_CT_, or pVSV eGFP dG-2S-del18 plasmids. **CRITICAL:** The transfected cells are transferred into a BSL2+ facility. 26. Incubate the transfected cells for 4 days. 27. Collect the supernatant and freeze-thaw cells 3 times to release all viruses from the cells. Freeze the combined fractions at −80°C freezer in aliquots. ***Note:*** Usually after 2–3 days, cells become rounded and detached due to virus replication and G protein expression in the cells. Cell syncytia can be also detected (Figure 3).

**CRITICAL:** The transfected cells are transferred into a BSL2+ facility.

26. Incubate the transfected cells for 4 days.
27. Collect the supernatant and freeze-thaw cells 3 times to release all viruses from the cells. Freeze the combined fractions at −80°C freezer in aliquots.

***Note:*** Usually after 2–3 days, cells become rounded and detached due to virus replication and G protein expression in the cells. Cell syncytia can be also detected (Figure 3).

28. **Day 5:** Seed 4 × 10^5^ BHK-21/WI-2 cells in a 6-well plate.
29. **Day 6:** Transfect the BHK-21/WI-2 cells with pCAGGS-G (0.4 μg/well) using Effectene according the manufacturer’s instructions (https://www.qiagen.com/us/products/discovery-and-translational-research/functional-and-cell-analysis/transfection/effectene-transfection-reagent/#orderinginformation). Cells that express the VSV-G from a separate plasmid support more efficient amplification of the rescued rcVSV in the subsequent step.
30. **Day 7:** Add the collected rescue viruses to the transfected cells and incubate for 3 days.

**CRITICAL:** Add all rescued viruses from step 27 to the cells. Do not discard the viruses after 1-h adsorption. Instead, add 1 mL of media to the existing media and incubate for a total of 3 days. At this step, no obvious cytopathic effects (CPE) are typically observed. It is not recommended to incubate the cells longer than 72 h as this will lead to a decrease in viral titer.

31. **Day 9:** Seed 4 × 10^5^ target cells which express the receptor for the recombinant rcVSVs in a 6-well plate and incubate at 37°C for 12–16 h. For HIV-1 Env and SARS-CoV-2 Spike displaying viruses, we use Cf2Th-CD4/CCR5 and 293T-ACE2 cells, respectively.
32. **Day 10:** Collect the virus from step 30 by centrifugation of the supernatants at 800 × *g* for 10 min at 4°C.
33. Serially dilute the collected viruses and add them to the 6-well plate containing the target cells that were prepared in step 31. Incubate for 2 h at 37°C to allow virus entry. Gently shake the plate every 15 min to evenly distribute the virus.
34. During incubation, prepare 2% low melting agarose by heating in a microwave for 30–45 s. Keep at 42°C in a water bath. We typically use SeaPlaque Agarose for cell culture experiments.
35. Discard the media after the 2-h incubation (step 33) and wash the cells 3 times with PBS.
36. Add equal volume of 2% low melting agarose, which was prepared in step 34, to 2× DMEM media and add 2 mL of the mixture to each well.
37. Incubate for 10–20 min at 20°C–25°C to allow agarose solidification and incubate the infected cells at 37°C for 2 days.
38. **Day 12:** Seed ∼3 × 10^5^ target cells/well in a 6-well plate. Target cells for HIV-1 env and Spike protein-expressing rcVSVs are Cf2Th-CD4/CCR5 and 293T-ACE2 cells, respectively.
39. **Days 13–15:** Collect the well-isolated plaques in the lowest dilutions by puncturing the agarose using a blue pipet tip. Transfer the plaque to 200 μL of DMEM and freeze and thaw the viruses 3 times by placing the tube on dry ice-methanol and then transferring the tube to a 37°C water bath. Each step of freeze and thaw should take at least 5 min. Add viruses to the target cells of each well of the 6-well plate (from step 38). Incubate the infected cells for 2–3 days at 37°C and monitor CPE.

**Figure 3:**
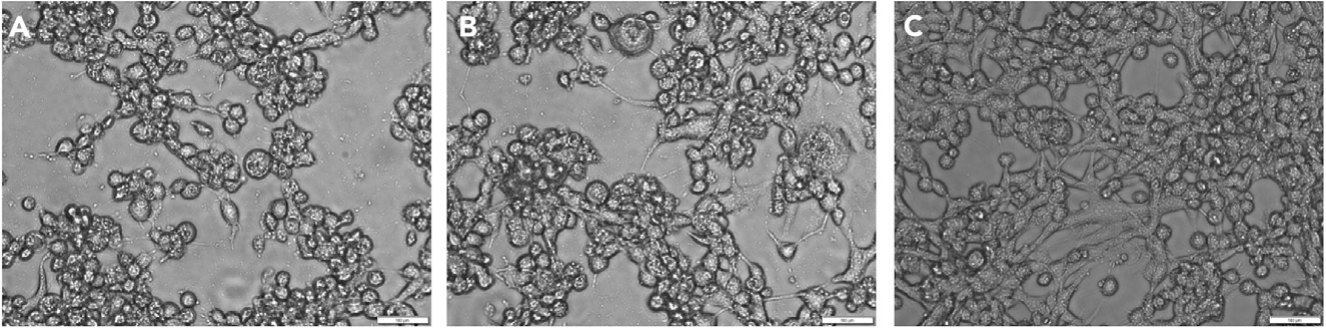
Cytopathic effect of rcVSVs in BHK-21/WI-2 cells 24 h post inoculation (A) rcVSV-AD8-M infected cells. (B) rcVSV-2S-Del18 infected cells. In (A) and (B), as a result of virus replication, the infected cells become rounded and detached from the surface, floating in the media. Some infected cells form syncytia 24 h post inoculation. Both rounded cells and cell syncytia of infected cells can be detected. (C) Uninfected BHK-21/WI-2 control cells. Scale bar, 100 μm.

### Viral passage

**Timing: 4 days**

Passage the viruses in the target cells for at least 3 times to increase the virus titer. Amplification efficiency can differ among Envs. For example, rcVSV-AD8-M titer significantly increased after 3 passages but rcVSV-SARS-CoV-2 spike, required 9 passages to reach a reasonable titer.

40. **Day 1:** Seed 1 × 10^6^ target cells expressing the receptor in T25 tissue culture flask.
41. **Days 2–4:** Add collected rcVSVs from step 39 to the T25 flask and incubate for 1 h at 37°C. Remove the virus and wash once with PBS.
42. Add 3 mL of media to each T25 flask supplemented by penicillin-streptomycin antibiotics. Incubate for no more than 72 h and monitor daily for the presence of CPE. Collect the rcVSVs when approximately 70% of the cells exhibit CPE.

### Assays to verify recombinant rcVSVs growth

**Figure 4:**
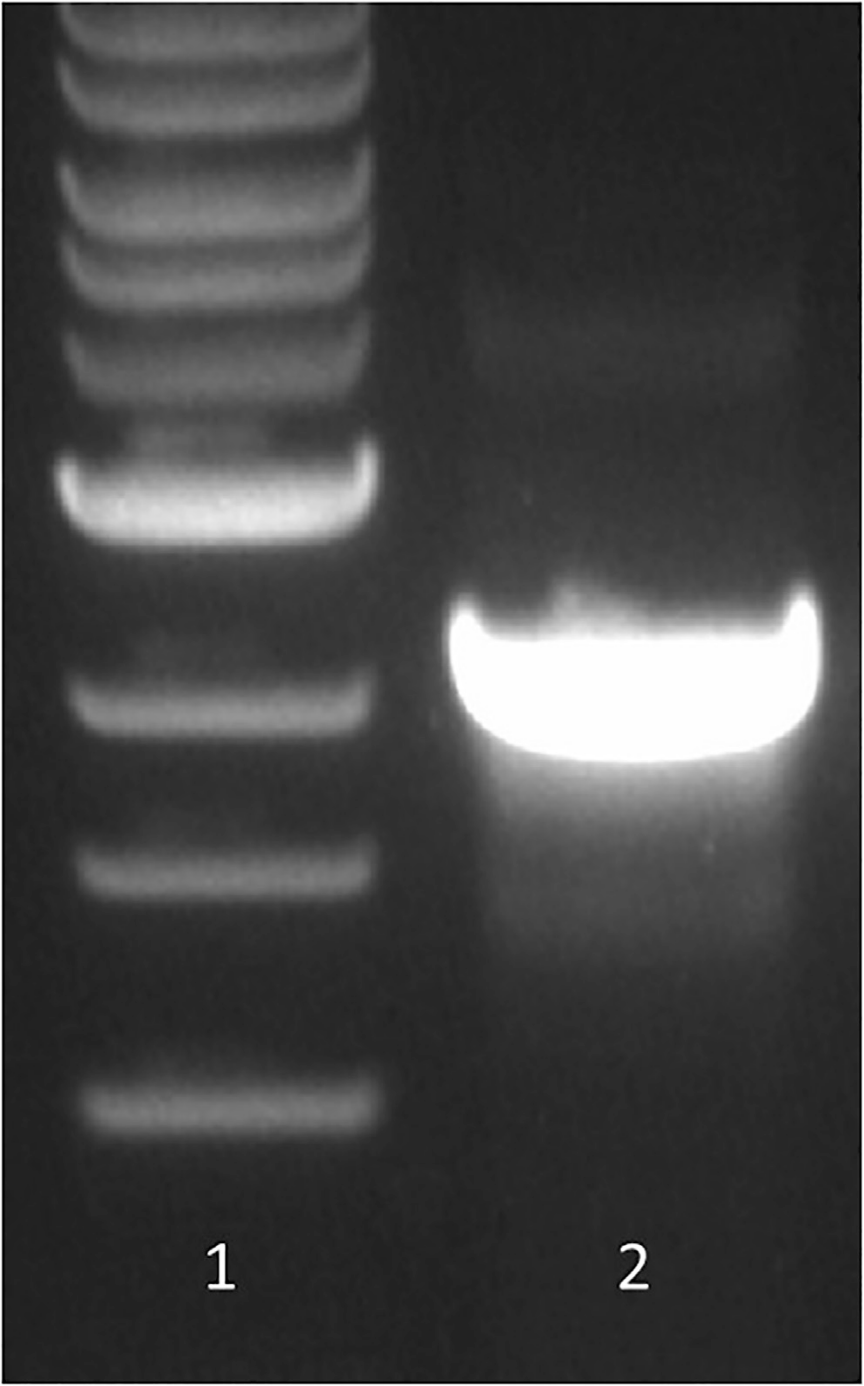
Assay to detect rcVSVs RT-PCR analysis of rcVSV-AD8M replication. Lane 1, DNA ladder; lane 2, rcVSV-AD8-M.

After final passage and before proceeding to further experiments, it is necessary to verify that the VSV genome contains the heterologous env gene by reverse transcription-PCR (Figure 4) and sequencing. The level of Env expression in target cells can be also analyzed by western blot or immunofluorescence assays.

### Reverse transcription polymerase chain reaction (RT-PCR)

**Timing: 1 day**

Viral RNA is extracted from supernatant of infected cells or directly from infected cells by a commercial kit according to the manufacturer`s instruction. We typically use the NucleoSpin RNA Virus Kit (Macherey-Nagel; https://www.mn-net.com/us/nucleospin-rna-virus-mini-kit-for-viral-rna-from-cell-free-fluids-740956.50) but many other kits are available from commercial vendors. The isolated viral RNA is used as a template for RT-PCR. Either specific primers or random hexamer can be used for the cDNA synthesis of the viral RNA by a reverse transcriptase.

43. Set up the following RT reaction by adding the materials in the order they are listed:

**Table:** 

50 ng random hexamer or specific primers	1 μL
10 ng total RNA	1 μL
dNTP Mix (10 mM each)	1 μL
Sterile, distilled water	11 μL

***Note:*** In our experience random hexamer worked better than specific primers for the RT-PCR reaction.

Heat mixture to 65°C for 5 min and quickly place on ice. Collect the contents of the tube by brief centrifugation and add:

**Table:** 

5× First-Strand Buffer (provided with the SuperScript II Reverse Transcriptase)	4 μL
0.1 M DTT	2 μL

incubate at 25°C for 12 min, add 1 μL (200 units) of SuperScript II RT and incubate at 42°C for 50 min. Inactivate the reaction by incubating at 70°C for 15 min.

44. PCR protocol for rcVSVs amplification

**Table:** 

Distilled water (dH_2_O)	40.5 μL
10× PfuUltra II reaction buffer	5 μL
dNTP mix (25 mM each dNTP)	0.5 μL
cDNA template (step 43 products)	1 μL
Forward primer (10 μM)	1 μL
Reverse primer (10 μM)	1 μL
PfuUltra II fusion HS DNA polymerase	1 μL

**Table:** Thermal cycle program:

**Initial Denaturation**	95°C 3 min	1× cycle
**Denaturation**	95°C 20 s	40× cycle
**Annealing**	60°C 20 s	
**Extension**	72°C 2 min^a^	
**Final extension**	72°C 5 min	1× cycle

a Elongation time depends on the DNA polymerase and insert length.

***Notes:***

PCR product can be directly sequenced, or it can be cloned into a plasmid and then single clones can be sequenced.

### Application of rVSVs displaying the SARS-CoV-2 spike

**Timing: 3–4 days**

As an example, for an application of rVSV, we describe the use of scVSV-2S-del18.luc for testing virus neutralization by serum of a mouse immunized with the soluble SARS-CoV-2 spike.

**Figure 5:**
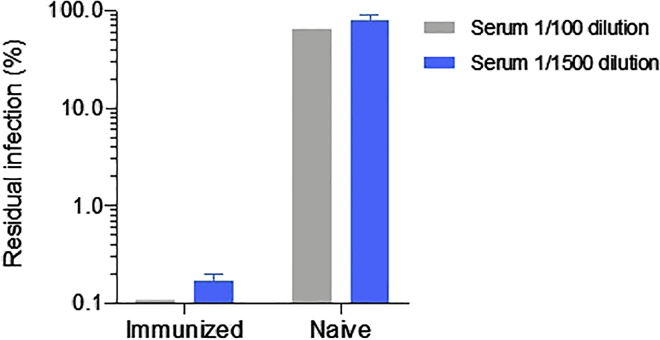
Sensitivity of scVSV-2S-del18.luc to serum of a mouse immunized with the soluble SARS-CoV-2 spike Experimental procedures are described in the section Application of rVSVs displaying the SARS-CoV-2 spike. Data are normalized to scVSV-2S-del18.luc control and are shown as mean ± SD of the readout from triplicate wells. All procedures were approved by the Institutional Animal Care and Use Committee (IACUC) of the University of Minnesota.

45. **Day 1:** Blood is collected from a BALB/c mouse that was subcutaneously immunized three times (with a two weeks interval between immunizations), each with 25 μg of soluble SARS-CoV-2 spike protein in 50 μL PBS (ectodomain, described in Wrapp *et al.* 2020) or from a BALB/c mouse immunized with PBS (naïve mouse). We mixed 50 μL of SARS-CoV-2 spike or PBS with 50 μL of AddaVax adjuvant (InvivoGen; cat# vac-adx-10) for all immunizations and all procedures are approved by the Institutional Animal Care and Use Committee (IACUC) of the University of Minnesota.
46. The serum is collected before each immunization and 2 or 3 weeks after the last immunization. The serum is separated from blood components by allowing the blood to clot for 30 min at 20°C–25°C and centrifugation at 1,000–2,000 × *g* for 10 min at 4°C. Transfer the top, clear fraction to a 1.5 mL tube.
47. Titer the scVSV-2S-del18.luc as described in steps 13–16 and determine a working dilution that results in a readout in the linear range of the titration curve.
48. Seed 2 × 10^4^ cells/well of 293T-ACE2 target cells in 96-well white luminometer plate.
49. **Day 2:** Make serial dilutions of the serum (e.g., 1:40, 1:200, 1:1,000, 1:5,000) in DMEM. Mix the diluted serum with equal volume of the scVSV-2S-del18.luc and incubated at 37°C for 1 h.
50. Add 100 μL of the serum-virus mixture to the 293T-ACE2 target cells, incubate the plate at 37°C for 6–20 h, and measure the luciferase activity as described in Ratnapriya et al. 2020 (Figure 5).

## Expected outcomes

Our protocol describes the procedures to produce scVSVs and rcVSVs. The expected outcomes are valuable reagents to study molecular virology and immunology. We demonstrate that scVSV-SARS-2-S del18 pseudoviruses expressing green fluorescence protein (GFP) are a valuable tool for studying virus entry. Virus infection can be estimated from the level of GFP expression in the target cells, which can be measured by flow cytometry (Figure 1). scVSV-2S-del18.luc viruses can be assayed in a 96-well format and therefore suitable for large scale measurements of virus entry and neutralization (Figure 5). In addition, the stock scVSV-G can be amplified by infecting cells that express the VSV-G protein from a separate plasmid and provide an infinite source to produce scVSVs displaying different Envs.

## Limitations

• Infection of rVSVs is efficient and robust. We and others have detected low levels of 293T infection by scVSV-2S-del18 even without exogenous expression of the ACE2 receptor in these cells. It is important to compare infection of target cells that express the related receptor with the same cells that do not express the receptor to evaluate the levels of receptor-independent entry. We also recommend using BHK-21/WI-2 cells for rVSV preparations.
• rcVSVs have to grow in cells for virus rescue and amplification. Initial amplification can be facilitated by expression the VSV-G from a separate plasmid (in trans) in cells but rcVSVs will then have to be serially passaged in target cells that express the related receptor until reasonable titer of the virus is detected. Thus, target cells must support robust replication of rcVSVs.

## Troubleshooting

### Problem 1

No colonies after Gibson assembly (step 21).

### Potential solution

• Make sure the Gibson assembly master mix is working by including positive controls. A positive control can be found in the NEBuilder kit.
• Toxic DNA sequence. Some sequences may be toxic to bacteria.
  ○ Include intron upstream to GOI.
  ○ Grow the bacteria at 20°C–25°C for 24–48 h.

### Problem 2

Low titer of pseudotyped scVSV (step 16).

### Potential solution

• Low transfection efficacy: Use positive control for transfection and test also other transfection reagents
• Incubate cells after transfection for 24–48 h before infecting with scVSV-ΔG-luc
• Increase the titer of the scVSV-ΔG-luc to allow more viruses to infect production cells.
• Collect the virus no more than 24 h post infection

### Problem 3

Low titer of rcVSVs (step 39).

### Potential solution

• We tried different ratios of plasmids for transfection and provide the optimal conditions for rcVSV production in our lab, but the plasmid ratio can be further optimized for each case.
• Titer may be low for collecting rcVSVs before day 3 or after day 5. After transfection of the plasmids (step 25) incubate for 3–5 days. Passaging rcVSVs in BHK-21 cells that express the VSV-G can increase the titer.
• If the titer after the initial passages are still low, you can continue passaging until you reach higher titer. For rcVSV-SARS-CoV-2 Spike, we obtained the high titer only after 9 passages in the target cells.
• Do not freeze-thaw the viruses for more than 3 times.
• Do not use trypsin for splitting the cells a day before virus inoculation. This may decrease the cell-surface expressed receptors and can prevent virus entry and replication.

## Resource availability

### Lead contact

Further information and requests for resources and reagents should be directed to and will be fulfilled by the Lead Contact, Alon Herschhorn (aherschh@umn.edu).

### Materials availability

Materials generated in this study are available upon request.

### Data and code availability

This study did not generate any unique datasets or code.

## References

[bib1] Bar K.J., Sneller M.C., Harrison L.J., Justement J.S., Overton E.T., Petrone M.E., Salantes D.B., Seamon C.A., Scheinfeld B., Kwan R.W. (2016). Effect of HIV antibody VRC01 on viral rebound after treatment interruption. N. Engl. J. Med..

[bib2] Baum A., Fulton B.O., Wloga E., Copin R., Pascal K.E., Russo V., Giordano S., Lanza K., Negron N., Ni M. (2020). Antibody cocktail to SARS-CoV-2 spike protein prevents rapid mutational escape seen with individual antibodies. Science.

[bib3] Bresk C.A., Hofer T., Wilmschen S., Krismer M., Beierfuß A., Effantin G., Weissenhorn W., Hogan M.J., Jordan A., Gelman R.S. (2019). Induction of Tier 1 HIV neutralizing antibodies by envelope trimers incorporated into a replication competent vesicular stomatitis virus vector. Viruses.

[bib4] Case J.B., Rothlauf P.W., Chen R.E., Kafai N.M., Fox J.M., Shrihari S., McCune B.T., Harvey I.B., Smith B., Keeler S.P. (2020). Replication-competent vesicular stomatitis virus vaccine vector protects against SARS-CoV-2-mediated pathogenesis. bioRxiv.

[bib5] Flemming J., Wiesen L., Herschhorn A. (2018). Conformation-dependent interactions between HIV-1 envelope glycoproteins and broadly neutralizing antibodies. AIDS Res. Hum. Retroviruses.

[bib6] Furuyama W., Reynolds P., Haddock E., Meade-White K., Quynh Le M., Kawaoka Y., Feldmann H., Marzi A. (2020). A single dose of a vesicular stomatitis virus-based influenza vaccine confers rapid protection against H5 viruses from different clades. NPJ Vaccines.

[bib7] Harris M., Ratnapriya S., Chov A., Cervera H., Block A., Gu C., Talledge N., Mansky L.M., Sodroski J., Herschhorn A. (2020). slow receptor binding of the noncytopathic HIV-2UC1 Envs is balanced by long-lived activation state and efficient fusion activity. Cell Rep..

[bib8] Herschhorn A., Finzi A., Jones D.M., Courter J.R., Sugawara A., Smith A.B., Sodroski J.G. (2011). An inducible Cell-Cell fusion system with integrated ability to measure the efficiency and specificity of HIV-1 entry inhibitors. PLoS One.

[bib9] Herschhorn A., Gu C., Espy N., Richard J., Finzi A., Sodroski J.G. (2014). A broad HIV-1 inhibitor blocks envelope glycoprotein transitions critical for entry. Nat. Chem. Biol..

[bib10] Herschhorn A., Ma X., Gu C., Ventura J.D., Castillo-Menendez L., Melillo B., Terry D.S., Smith A.B., Blanchard S.C., Munro J.B. (2016). Release of GP120 restraints leads to an entry-competent intermediate state of the HIV-1 envelope glycoproteins. mBio.

[bib11] Hoffmann M., Kleine-Weber H., Schroeder S., Krüger N., Herrler T., Erichsen S., Schiergens T.S., Herrler G., Wu N.H., Nitsche A. (2020). SARS-CoV-2 cell entry depends on ACE2 and TMPRSS2 and is blocked by a clinically proven protease inhibitor. Cell.

[bib12] Jia M., Liberatore R.A., Guo Y., Chan K.W., Pan R., Lu H., Waltari E., Mittler E., Chandran K., Finzi A. (2020). VSV-displayed HIV-1 envelope identifies broadly neutralizing antibodies class-switched to IgG and IgA. Cell Host Microbe.

[bib13] Jiang P., Liu Y., Yin X., Yuan F., Nie Y., Luo M., Aihua Z., Liyin D., Ding M., Deng H. (2006). Elicitation of neutralizing antibodies by intranasal administration of recombinant vesicular stomatitis virus expressing human immunodeficiency virus type 1 gp120. Biochem. Biophys. Res. Commun..

[bib14] Kapadia S.U., Simon I.D., Rose J.K. (2008). SARS vaccine based on a replication-defective recombinant vesicular stomatitis virus is more potent than one based on a replication-competent vector. Virology.

[bib15] Langlois R.A., Shapiro J.S., Pham A.M., tenOever B.R. (2012). In vivo delivery of cytoplasmic RNA virus-derived miRNAs. Mol. Ther..

[bib16] Lawson N.D., Stillman E.A., Whitt M.A., Rose J.K. (1995). Recombinant vesicular stomatitis viruses from DNA. Proc. Natl. Acad. Sci. U S A.

[bib17] Liberatore R.A., Mastrocola E.J., Cassella E., Schmidt F., Willen J.R., Voronin D., Zang T.M., Hatziioannou T., Bieniasz P.D. (2019). Rhabdo-immunodeficiency virus, a murine model of acute HIV-1 infection. eLife.

[bib18] McLellan J.S., Pancera M., Carrico C., Gorman J., Julien J.-P., Khayat R., Louder R., Pejchal R., Sastry M., Dai K. (2011). Structure of HIV-1 gp120 V1/V2 domain with broadly neutralizing antibody PG9. Nature.

[bib19] Pancera M., Zhou T., Druz A., Georgiev I.S., Soto C., Gorman J., Huang J., Acharya P., Chuang G.-Y., Ofek G. (2014). Structure and immune recognition of trimeric pre-fusion HIV-1 Env. Nature.

[bib20] Parrish N.F., Gao F., Li H., Giorgi E.E., Barbian H.J., Parrish E.H., Zajic L., Iyer S.S., Decker J.M., Kumar A. (2013). Phenotypic properties of transmitted founder HIV-1. Proc. Natl. Acad. Sci. U S A.

[bib21] Ratnapriya S., Chov A., Herschhorn A. (2020). A protocol for studying HIV-1 envelope glycoprotein function. STAR Protocols.

[bib22] Riva L., Yuan S., Yin X., Martin-Sancho L., Matsunaga N., Pache L., Burgstaller-Muehlbacher S., De Jesus P.D., Teriete P., Hull M.V. (2020). Discovery of SARS-CoV-2 antiviral drugs through large-scale compound repurposing. Nature.

[bib23] Rose N.F., Marx P.A., Luckay A., Nixon D.F., Moretto W.J., Donahoe S.M., Montefiori D., Roberts A., Buonocore L., Rose J.K. (2001). An effective AIDS vaccine based on live attenuated vesicular stomatitis virus recombinants. Cell.

[bib24] Schmidt F., Weisblum Y., Muecksch F., Hoffmann H.H., Michailidis E., Lorenzi J.C.C., Mendoza P., Rutkowska M., Bednarski E., Gaebler C. (2020). Measuring SARS-CoV-2 neutralizing antibody activity using pseudotyped and chimeric viruses. J. Exp. Med..

[bib25] Tani H., Morikawa S., Matsuura Y. (2012). Development and applications of VSV vectors based on cell tropism. Front. Microbiol..

[bib26] Wrapp D., Wang N., Corbett K.S., Goldsmith J.A., Hsieh C.-L., Abiona O., Graham B.S., McLellan J.S. (2020). Cryo-EM Structure of the 2019-nCoV Spike in the Prefusion Conformation. Science.

[bib27] Whitt M.A. (2010). Generation of VSV pseudotypes using recombinant DeltaG-VSV for studies on virus entry, identification of entry inhibitors, and immune responses to vaccines. J. Virol. Methods.

